# Tumescent TAPP: laparoscopic inguinal hernia repair after the preperitoneal tumescent injection of diluted lidocaine and epinephrine saline solution and carbon dioxide gas

**DOI:** 10.1007/s00595-016-1349-x

**Published:** 2016-05-10

**Authors:** Hiromi Tokumura, Ryohei Nomura, Fumito Saijo, Naoki Matsumura, Akihiro Yasumoto, Mitsuhisa Muto, Yu Katayose, Kennichi Takahashi, Sho Haneda

**Affiliations:** Department of Surgery, Tohoku Rosai Hospital, 4-3-21 Dainohara, Aoba-ku, Sendai, Miyagi Prefecture 981-0911 Japan

**Keywords:** Tumescent laparoscopic transabdominal preperitoneal hernia repair, Inguinal hernia, Tumescent local anesthesia

## Abstract

**Purpose:**

Laparoscopic transabdominal preperitoneal inguinal hernia repair (TAPP) is technically difficult and not infrequently followed by postoperative complications and pain, especially when performed by inexperienced surgeons. To simplify TAPP and reduce postoperative pain, we devised a novel procedure whereby TAPP is carried out after the inguinal preperitoneal infiltration of diluted lidocaine and epinephrine saline solution and carbon dioxide gas (tumescent TAPP). This report introduces the concept of tumescent TAPP and summarizes its operative results.

**Methods:**

About 120 ml of diluted lidocaine and epinephrine solution and 60 ml of CO_2_ gas were infiltrated into the inguinal preperitoneal space through a transabdominal needle before TAPP. Tumescent TAPP was performed for 400 patients (355 men, 45 women; mean age, 63.2 years).

**Results:**

Using tumescent TAPP, we found it easier to confirm the inguinal anatomy and dissect the preperitoneal layer and inguinal floor, with less bleeding. The mean operation time was 101.9 min and there were few perioperative complications and minimal pain.

**Conclusions:**

Tumescent TAPP makes conventional TAPP easier and safer; however, this procedure should be verified by a comparative study with conventional TAPP.

## Introduction

Laparoscopic inguinal hernia repair has emerged as an effective alternative to open mesh repair for inguinal hernias. In terms of less early postoperative pain, faster return to usual activities, and reduced chronic pain, meta-analysis results of randomized comparative trials have found it to be superior to open mesh repair [1–4]. However, it has a steeper learning curve than open mesh repair because of the level of technical difficulty [5–10]. In fact, laparoscopic hernia repair requires unique skills and understanding of the unfamiliar anatomy of the inguinal region, and serious complications or hernia recurrence can result if the surgeon is inexperienced [11, 12]. The main reasons for the procedure being technically challenging are the need for broad dissection of the thin and fragile peritoneum and the inguinal floor, including Cooper’s ligament, accompanying anatomical obscurity and bleeding to a greater or less extent during dissection, especially in laparoscopic transabdominal preperitoneal inguinal hernia repair (TAPP). To reduce the learning curve, we developed a new procedure called tumescent TAPP. The procedure involves performing TAPP after injecting a large amount of diluted tumescent analgesics and epinephrine [13–15] as well as carbon dioxide (CO_2_) gas into the preperitoneal space. Based on our experience of using this technique to treat 400 patients, we consider it to be technically easier and more reliable than conventional TAPP. This report describes our novel techniques named “tumescent TAPP” and summarizes our operative results.

## Materials and methods

Between June, 2011 and June, 2015, 400 patients underwent tumescent TAPP in our hospital (Table 1). There were 355 men and 45 women, with a mean age of 63.2 years (range 21–89 years).

**Table 1:** Baseline characteristics of the patients

Patients (*N*)	400
Age, years (range)	63.2 (21–89)
Male	354
Female	46
Unilateral hernia	346
Bilateral hernia	54
Recurrent hernia	20

### Technique

**Fig. 1:**
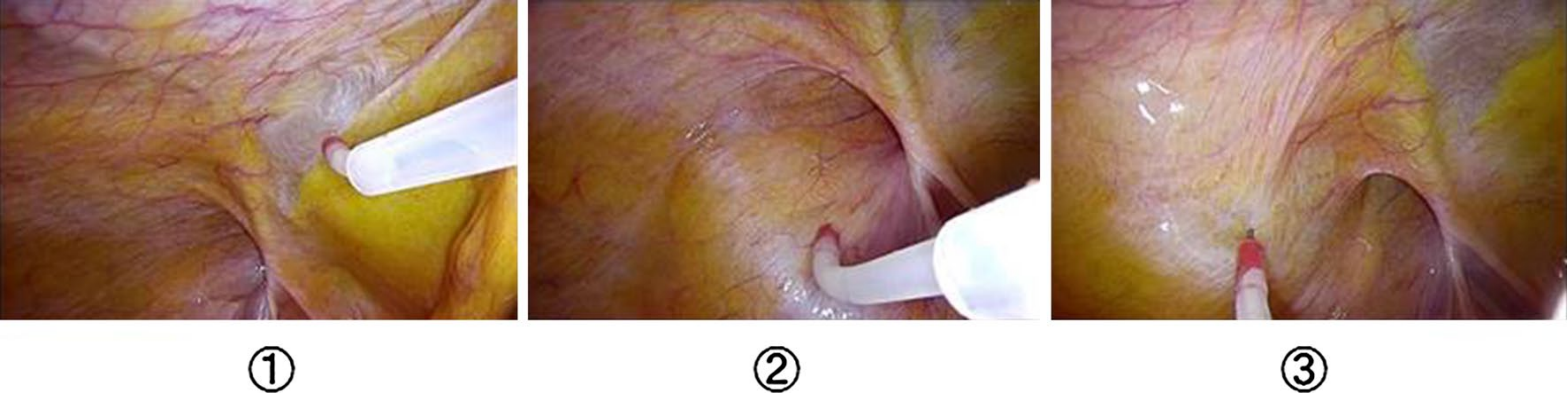
Preperitoneal tumescent method: using a ^®^Petineedle (Hakko Electric, Tokyo, Japan), we punctured the peritoneum in three places: ① medial to the inferior epigastric artery, lateral to the medical umbilical fold, and just ventral to Hesselbach’s triangle; ② at the lateral edge of the internal inguinal ring; and ③ ventral and lateral to the lateral triangle. The three punctures were made in this order, and 40 ml of the tumescent solution and 20 ml of CO_2_ gas were injected into the preperitoneal layer, respectively. This resulted in peritoneal swelling of the affected inguinal region

Under general anesthesia a 12-mm diameter trocar was placed in the supraumbilical region for the laparoscope. After establishing CO_2_ insufflation, two 5-mm trocars were inserted into the left and right rectus abdominis muscle margins at the height of the umbilicus. The diluted local analgesics and epinephrine (tumescent solution) and CO_2_ gas were injected to expand the area in the preperitoneal space in the affected inguinal region. The tumescent solution contained 0.2 ml (0.2 mg) of epinephrine, 30 ml (300 mg) of lidocaine hydrochloride, and 170 ml of physiological saline solution. A ^®^Petineedle (Hakko Electric, Tokyo, Japan) with an extension tube and three-way tap was inserted into the peritoneum from the trocar on the affected side. Using this needle, we punctured the peritoneum in three places: First, medial to the inferior epigastric artery, lateral to the medical umbilical fold, and just ventral to Hesselbach’s triangle; second, at the lateral edge of the internal inguinal ring; and third, ventral and lateral to the lateral triangle. The three punctures were made in this order, and 40 ml of the tumescent solution and 20 ml of CO_2_ gas were injected into the preperitoneal layer, respectively (Fig. 1).

**Fig. 2:**
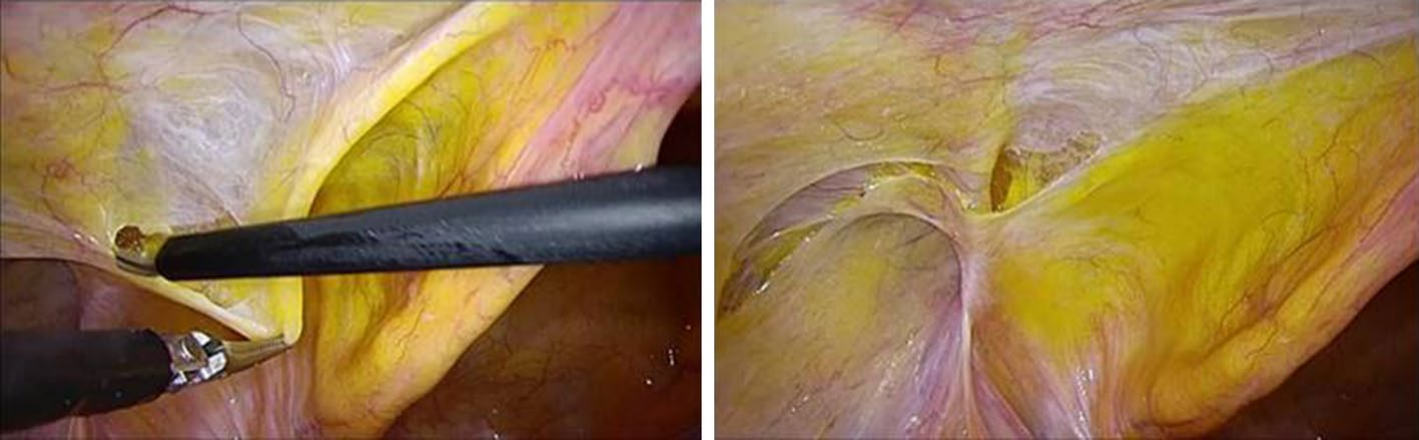
Peritoneal incision. The swelling caused the peritoneum to rise. The peritoneum was incised transversely through the ventral side of the inguinal area

**Fig. 3:**
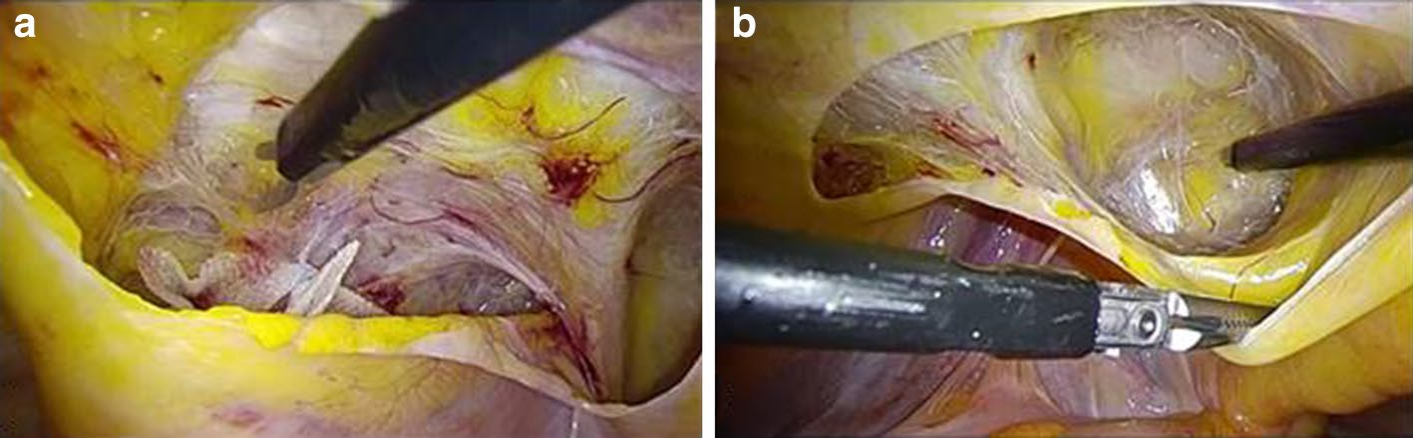
Preperitoneal layer dissection. **a** The preperitoneal layer was bluntly and sharply separated from the peritoneum to the dorsolateral and cranial side. **b** The dissection was continued to the medial side in the space of Retzius, in which clear infiltration of the tumescent solution can be seen on the ventral side of the prevesicular fascia. Cooper’s ligament, buried in the loose connective tissue, was thereby exposed easily with minimal bleeding

**Fig. 4:**
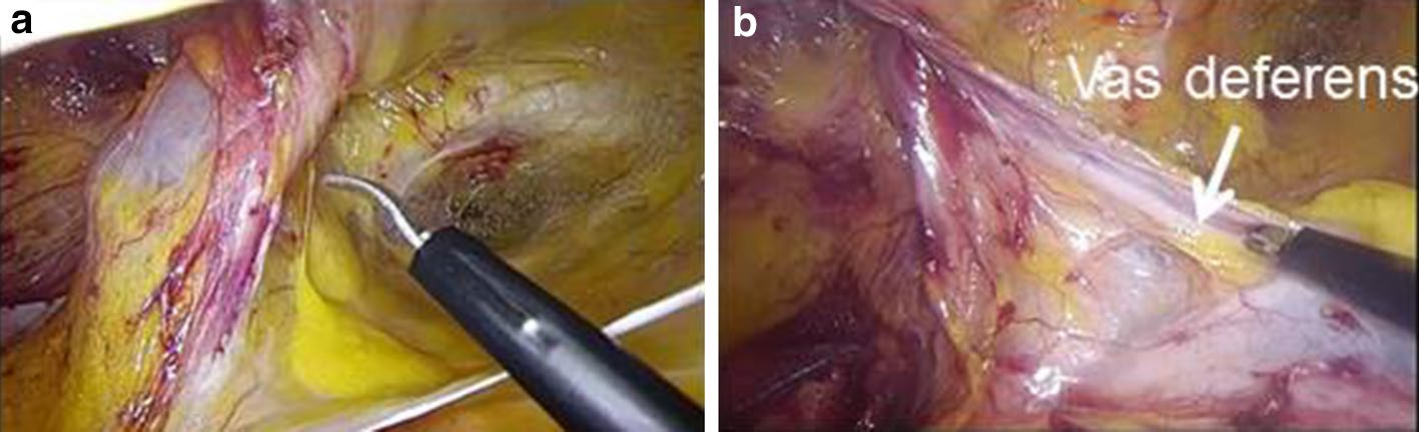
Hernial sac detachment. **a** The hernia sac was bluntly and sharply dissected on the ventral and lateral sides. **b** After the hernia sac was detached, the testicular vessels and vas deferens were parietalized

After the tumescent injection, the peritoneum rose to the surface, forming preperitoneal layer swelling (tumescence). Thereafter, in accordance with the conventional techniques of TAPP [9], the peritoneum was transversely incised above the internal inguinal ring (Fig. 2). The preperitoneal layer that had been already expanded with the tumescent solution was bluntly and sharply dissected from the peritoneum (Fig. 3). For an indirect hernia, we dissected the hernial sac at the ventral and dorsal side of the preperitoneal space, and then the testicular vessels and the vas deferens from the sac. After dissecting the entire circumference of the sac and confirming detachment of the posterior side, the sac was extracted or transected with monopolar scissors (Fig. 4). Total resection of the sac was not necessarily done. For a direct hernia, we dissected the prevesical space on the ventral medial side of the medial umbilical fold. The sac was pulled out from the ventral hernia orifice. As the tissue was dissected close to the abdominal wall, Cooper’s ligaments were exposed. Dissection was continued close to the midline. The vas deferens and testicular vessels were parietalized from the peritoneum and surrounding tissues. Finally, peritoneal dissection was completed in every direction. Taking the size of the mesh and hernia orifice and the degree of overlap into consideration, we confirmed that dissection was sufficient for the placement of mesh on the inguinal floor and that there was no bleeding. A piece of mesh 14–15 cm wide and 10–11 cm long was placed over the inguinal floor. Tacking was done at Cooper’s ligament, above the external triangle, on the dorsal side of the rectus abdominis muscle. The opening of the peritoneum was closed with running 3–0 absorbable sutures. After removing the trocars, the wounds were closed, and surgery was completed.

Our surgical team comprised seven surgeons, including three inexperienced surgical residents. For routine postoperative pain relief, patients were given two celecoxib 100 mg tablets a day for 3 days. The clinical path involved the patient being hospitalized the day before surgery and discharged 2 days after surgery. Follow-up outpatient examinations were conducted approximately 3 weeks postoperatively. If any patients complained of inguinal pain at their first outpatient visit after surgery, they were followed up regularly by the department until the pain resolved. The following surgical outcomes were investigated: (1) operation time, intraoperative bleeding, tumescent solution injection volume, surgical findings and general state; (2) postoperative pain and complications; and (3) pain and inguinal region findings after discharge.

## Results

### Intraoperative course

The hernia lesion was unilateral in 346 patients and bilateral in 54 patients. The mean operation time was 101.9 min for unilateral hernias and 143.6 min for bilateral hernias (Table 2). The mean volume of tumescent solution injected was 120–140 ml for the unilateral hernias. No intraoperative cardiovascular symptoms such as increased or decreased blood pressure or tachycardia were noted and no central nervous system symptoms or allergic reactions observed.

**Table 2:** Operative results

Patients (*N*)	400
Average operation time	
Unilateral hernia	101.9 min
Bilateral hernia	143.6 min
Bleeding volume	Little
Postoperative complications	
Seroma	12 patients (3 %)
Vas deferens injury	1 patient
Inferior epigastric artery bleeding	1 patient
Intestinal obstruction	1 patient
Average postoperative hospital stay	2.2 days (1–5 days)
Hernia recurrence	2 patients (0.5 %)

### Surgical findings

Tumescence resulted in swelling of the peritoneum, but there was no incidence of peritoneal rupture. The peritoneum was incised with minimal bleeding and tissue injury. It was easy to dissect and leave the preperitoneal layer, testicular blood vessels, and vas deferens in nearly all patients. The cobweb-like space of Retzius on the ventral side of the prevesical fascia and medial umbilical fold was identified and dissected with little difficulty due to the tumescent effect. Using scissor forceps, sharp dissection with monopolar electrocautery, which is not often used, was able to separate tissues in almost all patients. However, there was bleeding from the branch of the inferior epigastric artery and the vas deferens injury in one patient. There was no instance of bladder damage.

### Postoperative course

Subcutaneous bleeding around the trocar sites occurred in two patients, and a navel incision site infection developed in one patient. Most patients reported minimal postoperative pain. The mean postoperative hospital stay was 2.2 days. There were no hernia recurrences during the hospitalization period.

### Post-discharge course

One patient suffered intestinal obstruction 4 days after discharge, requiring emergency laparoscopic surgery to successfully release the adhesion of mesentery of the small intestine to the peritoneal suture site. Twelve patients (3 %) were found to have an inguinal seroma on the affected side about 3 weeks postoperatively, requiring fine-needle aspiration in nine to relieve a feeling of pressure. Fifteen patients (3.8 %) complained of pain at their first outpatient visit, and only three of these still complained of persistent slight pain 3 months after surgery. Despite the mild pain, these patients were actively involved in ordinary activities without the need for analgesics. Hernia recurrence developed in 2 of the 400 patients: 12 months after surgery in one and 35 months after surgery in the other.

## Discussion

TAPP has been reported to cause fewer postoperative complications and pain than open mesh repair [2, 3]. However, TAPP performed by inexperienced surgeons carries a risk of operative complications [11]. The conventional TAPP techniques are demanding and associated with a significant learning curve, and hernia recurrences are not uncommon when the operation is performed by unskilled surgeons [12]. The main technical challenges of TAPP are the difficulty to extensively dissect the peritoneum and inguinal floor, including Cooper’s ligament, around the medial umbilical fold, and the difficulty to identify the unfamiliar surgical anatomy during the dissection. To reduce the degree of these difficulties, we applied preperitoneal tumescent local anesthesia just before performing TAPP [16]. After establishing insufflation, a large amount of tumescent solution and CO_2_ gas was injected into the preperitoneal layer of the inguinal region through a needle catheter inserted via trocars. TAPP was then done in the usual manner. Using this new procedure, we expected to achieve advantages as with other procedures done with tumescent local anesthesia [13–15].

Originally, tumescent local anesthesia involved the injection of a large amount of diluted lidocaine and epinephrine solution during liposuction [13]. The hydrodissection effects of tumescence made it easier to suction fat, and the diluted lidocaine and epinephrine solution minimized bleeding and provided longer pain relief both intraoperatively and postoperatively. Moreover, the local analgesics had fewer toxic side effects than conventional local anesthesia. The subsequent application of tumescent local anesthesia in open hernia repair was found to reduce intraoperative bleeding, facilitate dissection through the separation of tissue planes by local analgesic infiltration (hydrodissection), and decrease postoperative pain as for the liposuction [17, 18]. Because the epinephrine in the tumescent solution not only inhibits bleeding through its vasoconstrictive effects, but also delays the absorption of local analgesia in systemic circulation, it greatly reduces the toxicity and side effects associated with local analgesics [13].

In the present study, 400 patients underwent TAPP after the injection of approximately 120 ml of tumescent solution and 60 ml of CO_2_ gas into the preperitoneal space around the inguinal region. The intraoperative findings suggested that hydropressure of the tumescent solution and CO_2_ gas pressure caused the tissue in the mainly preperitoneal space to expand, making it easier to visually confirm the anatomy of the peritoneum, preperitoneal space, and transverse fascia, and to perform the necessary dissection with little damage to other tissues. Furthermore, because there was minimal bleeding, sharp dissection was possible in almost all patients and hemostasis of the dissection layer was rarely necessary. Although there were concerns that the surgical field might submerge with the water invasion from the tumescent solution, we did not encounter this. In our experience of tumescent TAPP, the injection of 120 ml of the tumescent solution into the preperitoneal layer was appropriate to achieve tumescence. If less tumescent solution was less injected, the desired effects of the tumescent technique were not necessarily provided, whereas too much tumescent solution could damage the peritoneum or result in the side effects associated with these local analgesics.

CO_2_ gas was injected in addition to the tumescent solution because it allowed us to dissect the peritoneum and preperitoneal layer more efficiently than with only the tumescent solution [16]. Consequently the tumescence of the tumescent solution and CO_2_ gas facilitated dissection and preservation of the peritoneum and preperitoneal layer. Moreover, the tumescent solution infiltrated the prevesical space, making it easy to detect the anatomy and finely dissect the prevesical fascia without causing damage. Cooper’s ligament was also able to be easily exposed with less bleeding.

Conventional TAPP has been reported to be associated with less discomfort and pain in the inguinal region, both postoperatively and after hospitalization, than open hernia repair [1–4]). However, McCormack et al. [7] reported that 13.5 % of patients were left with chronic pain after TAPP, which is not insignificant [18–20]. In the present series, postoperative pain was generally minimal. Although a small number of patients complained of postoperative pain, it resolved within 3 months after surgery in all except three. The long acting effects of tumescent local anesthesia was assumed to be the reason for the minimal postoperative pain. Chronic pain was also thought to be rare. Accordingly, we consider that postoperative pain after tumescent TAPP would be less than after conventional TAPP, although a future competitive study is necessary and could demonstrate the effect.

No side effects associated with local analgesics and epinephrine were observed in this study. Even if tumescent TAPP is performed for bilateral hernias, it involves only a maximum dose of 60 mg of lidocaine hydrochloride, which is not considered enough to cause toxic or circulatory problems [12, 13]. Moreover, the CO_2_ gas injection into the preperitoneal space did not cause gas embolism or postoperative pulmonary complications. Uraoka et al. [21] reported submucosal elevation in terms of injecting CO_2_ gas for endoscopic submucosal resection (ESD) experimentally using porcine stomachs. The safety and efficacy of CO_2_ as a satisfactory submucosal injection agent during ESD have been demonstrated. Shikata H et al. [22] performed endovascular revascularization under CO_2_ gas angiography for patients with iodine allergy and renal dysfunction and found no direct or indirect complications of CO_2_ gas angiography after injecting up to 200 ml of CO_2_ gas. Consequently, we think that the infusion of CO_2_ gas into the preperitoneal space does not cause any clinical problems unless a very large volume of CO_2_ gas, probably over 200 ml, is injected directly into blood vessels.

In terms of postoperative complications, Bittner et al. [23] reported that hematoma and seroma developed in 4.2 and 4.4 %, respectively, of patients who underwent conventional TAPP. These complications appear to be less common after tumescent TAPP. There were also fears that the injection of a large amount of tumescent solution could cause rupture of the peritoneum or that the puncture needle could cause vascular damage. Although these events did not occur in the present study, their possibility should not be disregarded. The operation time was not thought to be shorter than that for conventional TAPP; however, since the surgeons in this study included three inexperienced residents, it is anticipated that the tumescent effects of tumescent TAPP could shorten the operation time as the surgeons gain experience. The present study did not compare the operative results of tumescent TAPP with those of conventional TAPP. Until 8 years ago, we routinely performed conventional TAPP using smaller mesh and a hernia stapler, but few patients have undergone conventional TAPP in the past 8 years. Therefore, we thought that it would not be meaningful to use the data on previous conventional TAPP cases to compare with the recent tumescent TAPP cases.

In conclusion, we devised a novel method of “tumescent TAPP inguinal hernia repair” in which a tumescent diluted local anesthetic solution including epinephrine and CO_2_ gas were injected into the inguinal preperitoneal space before the TAPP procedure. Favorable results were achieved in 400 patients who underwent this procedure. Thus, tumescent TAPP appears to offer technical and clinical improvements to conventional TAPP, although further comparative studies on this procedure are required.
